# Exploring the Potential of Multiomics and Other Integrative Approaches for Improving Waterlogging Tolerance in Plants

**DOI:** 10.3390/plants12071544

**Published:** 2023-04-03

**Authors:** Anshika Tyagi, Sajad Ali, Suvin Park, Hanhong Bae

**Affiliations:** Department of Biotechnology, Yeungnam University, Gyeongsan 38541, Gyeongbuk, Republic of Korea

**Keywords:** waterlogging, signaling, breeding, transgenic, multiomics, CRISPR-Cas, panomics

## Abstract

Soil flooding has emerged as a serious threat to modern agriculture due to the rapid global warming and climate change, resulting in catastrophic crop damage and yield losses. The most detrimental effects of waterlogging in plants are hypoxia, decreased nutrient uptake, photosynthesis inhibition, energy crisis, and microbiome alterations, all of which result in plant death. Although significant advancement has been made in mitigating waterlogging stress, it remains largely enigmatic how plants perceive flood signals and translate them for their adaptive responses at a molecular level. With the advent of multiomics, there has been significant progress in understanding and decoding the intricacy of how plants respond to different stressors which have paved the way towards the development of climate-resistant smart crops. In this review, we have provided the overview of the effect of waterlogging in plants, signaling (calcium, reactive oxygen species, nitric oxide, hormones), and adaptive responses. Secondly, we discussed an insight into past, present, and future prospects of waterlogging tolerance focusing on conventional breeding, transgenic, multiomics, and gene-editing approaches. In addition, we have also highlighted the importance of panomics for developing waterlogging-tolerant cultivars. Furthermore, we have discussed the role of high-throughput phenotyping in the screening of complex waterlogging-tolerant traits. Finally, we addressed the current challenges and future perspectives of waterlogging signal perception and transduction in plants, which warrants future investigation.

## 1. Introduction

Flooding is considered as one of the major constraints on crop productivity since it inhibits growth, delays planting, lowers vigor, and makes crops more susceptible to diseases and pests [1]. The incidence and frequency of flooding have significantly increased across the globe as a result of the drastic and rapid changes in global climate which caused remarkable impairment to crop production. Additionally, as a result of anthropogenically driven climate change events, such as a rise in the frequency of heavy precipitation and tropical cyclone activity, flooding events are projected to occur more frequently and with greater intensity around the globe [2,3]. Floods caused over two-thirds of all crop loss and devastation worldwide between 2006 and 2016, resulting in significant production losses [4]. Over 17 million km^2^ of agricultural land (10–20%) are expected to be affected annually by waterlogging with annual losses of more than $74 USD billion [5,6]. According to geographical reports, moist places such as the majority of tropical and subtropical zones may face waterlogging as a result of global warming due to the increase in both precipitation and evaporation. According to Rentschler et al. [7], flood disasters have an impact on 23% of the world’s population and have been steadily getting worse across all continents since the 1950s. The primary causes of waterlogging in agricultural systems include unpredictably strong rainfall, inadequate drainage systems, and soil types [8,9]. Depending on their magnitude, losses due to flooding range from 30% to 100%. However, the severity of flooding stress depends on the type of crop species, developmental stages, tissues exposed, and its duration. Together with the direct impact, flooding also worsens plant survival through various abiotic stress factors, such as decreased light availability, hypoxia, nutrient depletion, and changes to the physical and chemical composition of the soil. In addition, during flooding, plants are more vulnerable to pests and diseases, which further negatively, affect their growth and development. For instance, flooding increases Phytophthora blight and Fusarium wilt susceptibility in pigeon pea plants, resulting in severe yield losses [10]. Previous studies have shown the effects of waterlogging on important crops, such as maize, barley, rice, and legumes, and how it lowers their overall productivity. To maintain agricultural output in places vulnerable to floods and submersion because of heavy rainfall and poor drainage, waterlogging-tolerant cultivars must be developed. Likewise, to target distinct tolerance-related traits and design new varieties with higher waterlogging tolerance, researchers must have a thorough understanding of the intricate mechanisms underlying waterlogging tolerance. In this context, harnessing the potential of multiomics can provide novel insights for not only deciphering the molecular mechanism of waterlogging signaling cascades but also aids in the identification of potential targets that can be further utilized for developing long-lasting, stress-tolerant cultivars. 

With the advent of high-throughput sequencing tools, there has been a significant advancement in plant biology due to the availability and accessibility of references genome of both model and crop plants. These tools offer enormous potential for the identification of potential genes and mechanisms underlying the major agronomic features. Various omics approaches, such as genomics, transcriptomics, proteomics, metabolomics, ionomics, and phenomics, have played a key role in decoding the genetic and molecular basis of crop developmental and stress-associated traits. These studies led to the identification of potential genes, proteins, metabolites, and ions and their role in plant signal perception and transduction. For example, the application of multiomics in major crops, such as rice, wheat, maize, soybean, tomato, barley, and cotton, has played a key role in identifying potential components and decoding their role in different plant traits, such as growth, senescence, seed development, and stress tolerance [10,11,12,13,14,15]. Using mutagenomics and functional genomics, numerous mutants with distinctive variants in terms of stress tolerance, growth, and development have been discovered in a number of crops [11]. Many abiotic stress-tolerant crop phenotypes have been identified using integrative multiomics approaches [16]. The combination of omics with genome-wide association analysis (GWAS) has proven to be an effective method for analyzing the biochemical and genetic mechanisms of different traits in a number of model crop species, such as rice, maize, and tomato [17,18]. In addition, combining GWAS with omics, such as transcriptomics (eQTLs), proteomics (pQTLs), and metabolomics (mQTLs), may enable the discovery of novel genes and functional pathways in plants underpinning complex characteristics [19]. For example, Wen et al. [20] found that integration of metabolomics mQTLs and transcriptomics (eQTLs) led to identification of potential metabolites linked with kernel weight in *Glycine max.* Multiomics has significantly advanced breeding programs, and numerous QTLs/gene networks have been identified associated with stress tolerance [21]. Recently, panomics has emerged an elegant platform to integrate the complex omics data in order to develop a model for predicting complex traits [22,23]. For developing elite-resistant cultivars, integration of panomics with other environmental platforms can be utilized in conjunction with numerous data integration and functional genomics to find genes, QTLs, and markers [19]. There have been several tools, such as PAINTOMICS and COVAIN KaPPA-view, for analyzing multiomic data which will provide in silico-based validation before functional interpretation. Moreover, a strategy for the development of precision breeding has been presented that involves the integration of panomics and genome-editing techniques, such as CRISPR/Cas9 and TALENs [19]. These studies highlight the importance of omics tools in deciphering the molecular basis of diverse plant-traits-related growth and stress tolerance. Although there has been a significant advancement in decoding plant responses to abiotic stressors such as drought, heat, and salinity, there is limited information on how plants undergo transcriptomic, metabolomic, and translational reprogramming during waterlogging stress. Waterlogging is a complex process that causes transcriptional and translational changes in genes and proteins involved in the metabolism of carbohydrates, fermentation, photosynthesis, proteins related to stress, enzymes for ROS scavenging, and hormonal control. Many physiological, biochemical, molecular, and metabolite changes lead to plant adaptation from environmental stress via translational and posttranscriptional modification. Basic data on screening methods, physiological processes, and genetic traits are abundant in the literature related to waterlogging stress tolerance in plants. This information has been used for improving the efficiency of selection by accurate phenotypic and genotypic analysis using advanced genetic methods with massively parallel parameters. However, there is a limited understanding of flood signal perception and transduction in both model and crop plants. In this review, we have presented a general overview of the route from the available genomic resources, such as transcriptomics, proteomics, and metabolomics, to new emerging gene-editing tools that will be useful to identify novel stress tolerance genes for sustainable crop production. Furthermore, we have also highlighted the role of improved technologies, i.e., integrated omics and panomics, which would contribute to the production and advancement of breeding programs, benefiting from a budget-effective, environment-friendly, and, above all, less time-consuming approach.

## 2. Flooding Affects Diverse Crop Traits

**Figure 1:**
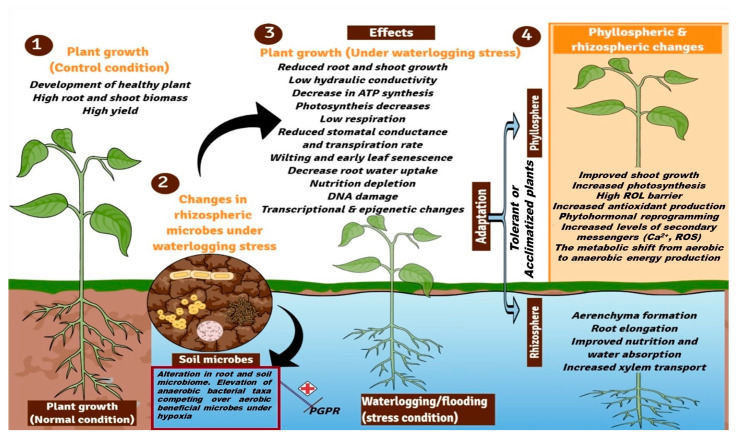
A general overview of the effect of waterlogging stress on sensitive plants and the adaptive mechanisms found in tolerant or acclimatized plants: (1) plant growth at normal/control conditions, (2) changes in rhizospheric microbes such as plant-growth-promoting bacteria (PGPR), (3) effect of waterlogging stress on plant morphophysiological, biochemical, and molecular changes, and (4) adaptation mechanisms in waterlogging-tolerant/acclimatized plants at both phyllospheric and rhizospheric regions in plants during waterlogging stress condition. ROL (radial oxygen loss); ROS (reactive oxygen species).

Flooding has a severe impact on different aspects of the crop, i.e., physiological, biochemical, and anatomical traits, as well as on overall plant performance (Figure 1). However, the impact of waterlogging stress in plants largely depends on cultivar type, provenances, timing and duration of waterlogging, and soil type. Oxygen depletion or hypoxia is the direct effect of flooding on crop physiology as it alters plant metabolism and causes an energy crisis. Lack of soil aeration during flooding causes a switch in from an aerobic (TCA cycle) to anerobic energy metabolism in the roots, which results in the accumulation of poisonous metabolites, such as ethanol, lactic acid, aldehydes, and oxygen radicals (ROS, H_2_O_2_), all of them promote early senescence and cell death [24,25].

Additionally, hypoxia not only inhibits aquaporin activity but also affects root metabolism, which prevents the growth and extension of lateral roots. For example, flood-altered aquaporin activity leads to the accumulation of ABA which leads to the closure of stomata which in turn affects the leaf growth. During flooding, stomatal closure confines carbon dioxide (CO_2_) diffusion to the chloroplasts and alters the net CO_2_ assimilation [26,27,28]. Flooding also inhibits the photosynthesis process by photoinhibition and reduced chlorophyll pigments. In addition, photosynthesis is also affected in plants by the reduction in nitrogen uptake and assimilation during waterlogging [29]. The accumulation of gaseous hormone ethylene (ET) during flood-induced hypoxia also alters auxin-mediated root responses thereby inhibiting key root-to-shoot functional traits [30]. Higher accumulation of ET in plants during waterlogging leads to leaf epinasty, induces stomatal closure, and reduces leaf growth [27,31]. Plants exposed to waterlogging cannot survive longer due to the accumulation of toxic substances, carbon starvation, cytoplasmic acidification, or disease outbreaks [29,32]. Changes in root function quickly affect the aerial components, with hydraulic and chemical signals predominating root-to-shoot transmission. Furthermore, we have summarized the effects of waterlogging on different plant traits in different crop systems in Table 1.

Waterlogging-induced alteration in plant’s physicochemical, anatomical, and soil chemistry directly affects its microbiome [41]. In nature, plants are nourished by their beneficial microbiome both under normal and stressful conditions. For example, plant microbiome provides incredible benefits to their hosts, such as stress resilience and nutrient availability, and promotes growth [42,43,44]. Growing evidence points to the microbiome as a crucial factor in plant health and resistance to flooding stress. Like other abiotic stress, flooding also alters plant microbiome and its functions. For instance, the alteration in the crop’s physiological and biochemical traits, as well as physio-chemical properties, by flooding causes a massive shift in microbial structure. There are several studies on rice plants that demonstrate how flooding affects the microbiome mostly in terms of bacteria, but archaea, oomycetes, fungi, and viruses remain largely unexplored. There are reports which have shown that flooding alters rhizospheric and bulk soil microbial communities [45,46,47]. Previous studies have revealed the effect of flooding on the rice phyllosphere microbiome and identified Firmicutes (54%) and Bacillus (52.63%) as the dominant taxa in flooded rice plants. According to Li et al. [48], the amount and length of flooding cause plants to experience a decline in the colonization of microbial endophytes. Similarly, *Myricaria laxiflora*, a riparian shrub that often experiences intermittent summer floods, had a lower endophyte diversity under anerobic circumstances [49]. Similar to this, flooded rice roots showed lower bacterial diversity [50]. Although there are numerous studies on how different biotic and abiotic stress changes plant microbiome structure, many drivers have been identified. However, how flooding stress alters microbiomes is least studied. For example, the role of root exudate chemistry, soil chemistry, host and microbiome signaling molecules, and sensors that drive microbiome assembly or alterations is not fully understood. However, hypoxia is considered as a major factor in flood-mediated alteration of the plant microbiome. Hypoxia constraints both plant and microbiome functionaries, for example, it inhibits root aerobic respiration, nutrient uptake, hydraulic conductance, plant growth, and development as well as enriches anaerobic microbes which cause detrimental effects on the host by their pathogenic nature and soil denitrification. Hence, there is a need to explore the potential of multiomics approaches to identify key drivers of flood-induced, host-mediated microbiome assembly. In order to reduce flooding stress in sensitive species and ecosystems, it is essential to understand how floods affect plant physiology and plant-associated microorganisms. Furthermore, we have highlighted the effect of waterlogging stress on plant-beneficial microbiota in (Figure 1).

## 3. Adaptive Responses of Plants against Waterlogging Stress

The majority of plants are vulnerable to waterlogging because it considerably reduces the rates of O_2_ and CO_2_ diffusion in the roots and stems of plants and significantly inhibits both photosynthesis and respiration. However, there are many reports that have highlighted the adaptive responses (morphological, biochemical, physiological, and anatomical) in plants against waterlogging stress (Figure 1). The primary morphological and anatomical alterations include the development of adventitious roots (ARs) or other aeration tissues, radial oxygen loss (ROL) barriers, rapid apical meristematic tissue extension, and the creation of air films in the upper cuticle [8,10,31]. Among them, the most important adaptive response against waterlogging stress is the formation of ARs. Interestingly, AR formation can, to some extent, substitute primary roots that perish due to hypoxic stress, preserving metabolic cycles and facilitating typical growth and development [51,52]. In comparison to the primary roots, the newly formed ARs have greater aerenchyma, which improves O_2_ uptake and diffusion capacity [53]. Another defense mechanism against waterlogging is the fast extension of plant apical meristems. It has been reported that the rapid elongation of tender stems and internodes aids to escape hypoxic conditions also called low oxygen escape syndrome (LOES) and quickly reaches the aerial environment, thereby maintaining a normal respiration [54]. Two important hormones ET and gibberellins are known to play a crucial role in the formation of plant apical meristems under waterlogging stress [54]. In plants, metabolic reprograming is another important adaptive response during waterlogging stress. For instance, the activation of the glycolytic and fermentation pathways, which induce the metabolic change from aerobic to anaerobic, is essential for maintaining an energy supply in plants under waterlogging stress [55,56]. At the cellular level, the activation of antioxidant enzymes and nonenzymatic antioxidants is another important adaptive role in alleviating the detrimental effects caused by oxygen radicals, such as ROS and H_2_O_2_, during waterlogging stress [57,58]. Previous studies have reported that higher levels of antioxidants, such as ascorbate peroxidase (APX), superoxide dismutase (SOD), peroxidase (POD), and catalase (CAT), in different crops were related to higher waterlogging tolerance [59,60]. Furthermore, we have also discussed the role of different hormones, such as ET, abscisic acid (ABA), auxin (AUX), salicylic acid (SA), jasmonic acid (JA), gibberellic acid (GA), and brassinosteroid (BR), in plant adaptive responses against waterlogging in Section 4. Although there have been numerous reports on different adaptive responses in both waterlogging-sensitive and -tolerant cultivars, there are many knowledge gaps in understanding the molecular mechanism of these complex traits. Future research in this area should concentrate on using multiomics and cutting-edge gene-editing tools to decode the complexity of molecular adaptive responses and to identify the key gene networks, paving the way for the introduction of these novel adaptive traits or genes to create waterlogging-resistant crops.

## 4. Waterlogging-Mediated Signaling Mechanism in Plants

**Figure 2:**
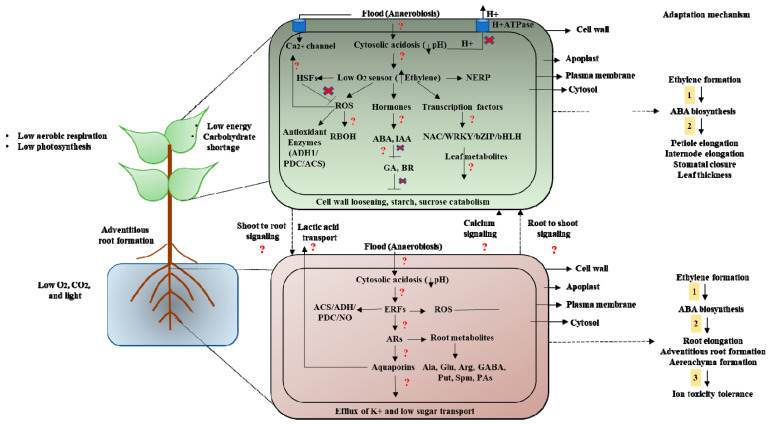
Illustration showing the root and shoot signaling as well as their adaptation mechanism during waterlogging or flooding stress condition in plants. Here, we have highlighted the signal perception and transduction changes in various compartments of the plant system with a focus on cell wall, plasma membrane, and cytosolic alterations. Furthermore, we have also highlighted how plants deal with long-distance bidirectional signaling, i.e., from root to shoot or shoot to root during waterlogging stress condition. Furthermore, we have shown various signaling players, such as ROS, calcium, ET, ABA, IAA, GR, and BR, in modulating the expression of various defensive genes, *alcohol dehydrogenase 1* (*ADH1*), *pyruvate decarboxylase* (*PDC*), and *1-aminocyclopropane-1-carboxylate synthase gene* (*ACS*), or transcriptional factors, such as *ERFs*, *HSPs*, *NAC*, *WRKY*, *bZIP*, and *bHLH*. Role of nitric oxide (NO), polyamines, viz., putrescine (Put) and spermine (Spm), amino acids, such as alanine (Ala), glutamic acid (Glu), and arginine (Arg), and gamma-aminobutyric acid (GABA) is also mentioned. We also highlighted different signaling pathways such as triggers by ET, ABA, and AUX for the formation of ARs and aerenchyma that leads to waterlogging tolerance.

How plants sense waterlogging signals and translate them into biochemical responses is not fully understood despite the availability of high-throughput tools. For example, the role of cell wall receptors and plasma membrane ion channels, ATPase, ROS, nitric oxide (NO), and hormonal cross-talk during hypoxia is not fully understood. In the last 10 years, there have been numerous studies which have highlighted the role of various players in waterlogging stress. For example, previous studies have shown that waterlogging triggers plant cell wall modifications or cell wall thickening by increased levels of hemicellulose and lignin deposition and also by the increased activity of xyloglucan endotransglucosyltransferace/hydrolase (XTH) and expansin (EXP) proteins, which highlights the importance of cell wall signaling in waterlogging stress. Cell wall modification is generally considered as one of the important traits of cell-wall-mediated signaling during abiotic and biotic stressors. Various cell wall receptors, such as wall-associated kinases (WAKs), Lectin receptor kinases (LecRKs), and Leucine-rich extensin proteins, have been identified as important cell-wall-based sensors for early signal perception and transduction. These sensors modulate an array of signaling cascades triggered by ROS, H^+^ATPase, calcium, and hormones. However, their role in waterlogging stress signal perception and transduction is largely unknown. Hence, future studies should be focused to decode the role of cell wall receptors in waterlogging signal perception that can provide novel insights for developing waterlogging-resistant cultivars. Calcium signaling has become a hallmark of decoding plant stress responses against stressors. During waterlogging stress, plants suffer oxygen depletion which triggers an array of signaling events, such as ROS formation, calcium burst, and hormonal reprogramming. Previous studies have shown that the role of ROS in activating calcium-dependent genes, such as *DH1*, *ERF73*, *HSP18.2*, *HsfA2*, *MYB2*, *PDC1*, *SUS1*, and *LDHSUS4*, involved in waterlogging stress tolerance. During hypoxia, calcium burst has emerged as an important early signaling player that modulates key metabolic and adaptive traits. However, it remains largely unknown which calcium channels are involved in waterlogging sensing. There are different calcium channels in plants, such as glutamate receptor-like channels (GLRs), cyclic nucleotide-gated channels (CNGCs), two pore channel 1 (TPC1), reduced hyperosmolality-induced [Ca^2+^] I increase 1 (OSCAs), MscS-like (MSL), and mid1-complementing activity (MCA), which play a vital role in plant stress signal transduction. The physiological mechanism of waterlogging tolerance via regulating endogenous hormonal levels during waterlogging stress is one of the key traits in plants. The role of plant hormones such as ET and auxin has been well studied under waterlogging stress. For instance, ET and AUX play important role in root modification as well as root-to-shoot communication during waterlogging stress. A key response of plants to waterlogging is the rapid buildup of ET which regulates an array of traits, such as adventitious root formation and ROS-induced aerenchyma formation [61,62]. Various ET transcription factors such as *ERF73/HRE1* are known to modulate the expression of various genes related to hypoxia stress tolerance [63]. It is well documented that *ERF-VIIs* transcription factors are crucial for the transmission of ET signals and the responses of plants to waterlogging stress [64,65]. For example, the maize gene *ZmEREB180*, a member of the ERF-VII family, positively controls the development and proliferation of ARs as well as the level of ROS and its overexpression increases survival during prolonged waterlogging stress [66]. Another important member of the ERF family is the rice *Sub1A* (Submergence 1A) gene which also leads to waterlogging stress tolerance by activating the expression of various hypoxia-tolerant genes. In addition to ET, ABA has also emerged as an important hormone in regulating waterlogging signaling traits in plants. For instance, ABA negatively regulates AR and aerenchyma formation during waterlogging conditions [67]. However, ABA positively regulates stomatal closure, thereby decreasing water loss from transpiration, and promotes waterlogging stress tolerance [68]. Auxin also plays a vital role in waterlogging tolerance in plants. Under waterlogging conditions, initial high levels of ET promote the production of AUX, which in turn not only inhibits ET biosynthesis but also triggers AR formulation by promoting cell division. Previous studies have revealed that exogenous treatment of AUX transport inhibitor in tobacco [69], cucumber [70], and tomato [71] inhibits AR formation during waterlogging stress. Many studies have revealed the role of SA and JA in waterlogging stress tolerance. For example, the exogenous application of SA in peach trees confers waterlogging stress tolerance by increasing the activity of various antioxidant enzymes and also the levels of proline which alleviate the detrimental attributes induced by waterlogging [72]. On the other hand, JA treatment greatly reduced waterlogging-related damage to soybean plants and enhanced plant growth [73]. Interestingly, during waterlogging stress, the interaction between JA and ET is crucial for the formation and growth of the root system and aerenchyma. Previous studies have shown that JA treatment increases ET content which further alleviates the detrimental effects of waterlogging stress [74]. Huang et al. [75] reported that exogenous application of GA enhances growth and stress trails peanuts during waterlogging stress. On the other hand, BR has also emerged as a key modulator of waterlogging signaling in plants. Previous studies have reported that the application of 24-epi-brassinolide (EBR) alleviated hypoxia-induced effects in cucumber seedlings by activating antioxidant enzymes, thereby reducing ROS [76]. Similarly, another study has shown that BR treatment enhanced AR formation, thereby improving oxygen supply which increases plant tolerance to hypoxia stress [77]. Nevertheless, these studies provided incredible information on the role of different hormones in waterlogging stress resilience. However, how their cross-talk fine-tunes plants’ response to waterlogging stress remains largely unexplored. Additionally, how these hormones balance stress and growth tradeoffs during waterlogging stress warrants future investigation. Further research is needed to determine how cell wall sensors and plasma membrane ion channels influence downstream signaling cascades during waterlogging stress, as well as how they control signal reception and transduction. In this context, the integration of multiomics, high-throughput phenotyping, and genotyping needs to be implemented to decode the intricacy of waterlogging signaling in plants which can offer new ways for improving waterlogging stress resilience. In this review, we have made one model highlighting the important signaling cascades and players involved in waterlogging signaling and also highlighting missing links (Figure 2). This model shows root- and shoot-driven signaling after waterlogging stress. 

## 5. Strategies for Improving Waterlogging Tolerance in Plants: Past, Present, and Future

With the advent of high-throughput sequencing technology, it became easier for plant researchers to identify genes and genetic regions that are linked with traits of interest. In the past, the application of multiomics and other high-throughput tools has been widely used in deciphering the molecular mechanism of stress tolerance or sensitivity in various crops during a number of biotic and abiotic stressors, which has significantly increased our understanding of plants’ response that paved the way for the development of stress-tolerant cultivars. Our knowledge of the mechanisms behind waterlogging stress signaling has been hampered by the paucity of investigations on the involvement of multiomics in plants under waterlogging stress, particularly in sensitive cultivars. In this review, we discussed the classical and novel approaches for developing waterlogging stress tolerance cultivars. 

### 5.1. Past: Classical Breeding and Genetic Engineering Approaches Used for Waterlogging Tolerance in Plants

Flooding tolerance in plants is a complex process which relies on both host and environmental factors, such as temperature, plant development stage, nutrition, soil type, and subtopography [78,79]. For this reason, it is essential for plant breeders to recognize and choose the best trait based on the stage of the plant’s development and environmental factors that could provide a more effective way of generating waterlogging-tolerant cultivars in regions with heavy rainfall or limited drainage. One of the key indicators utilized by researchers in several crops, including wheat (*Triticum* spp.) [80,81,82,83], soybean (*Glycine max*) [84], and barley (*Hordeum vulgare*) [85], is leaf chlorosis following waterlogging. In common wheat (*Triticum macha*) [81], Makha wheat (*Triticum macha*) [86], and maize (*Zea mays* ssp. mays) [87], it has been discovered that waterlogging tolerance trait (leaf chlorosis) is regulated by a single dominant gene, while in barley, it is a quantitative feature mostly regulated by additive genetic variation [88]. As leaf chlorosis had a moderately high heritability [88] and early-generation selection could be effective, accurate assessment of this characteristic still requires well-controlled waterlogging circumstances. Breeders find it very challenging to manage the numerous confounding environmental conditions in a field experiment including thousands of varieties. In this context, developing molecular markers linked to waterlogging resistance and marker-assisted selection (MAS) could be an alternative way for developing flood-tolerant cultivars. Finding the genetic bases of variation for key economic variables has been made possible through QTL analysis which may provide useful information for further genetic studies [89]. In addition, QTLs associated with important traits (e.g., leaf chlorosis, plant survival, and biomass reduction) have advanced significantly to enhance plant breeding for waterlogging tolerance (polygenic traits) using marker-assisted selection (MAS) in addition to the traditional field selection. Various studies have been conducted to find QTLs for flooding resistance-related traits, such as leaf senescence, leaf injury, plant survival, shoot elongation, root growth, and adventitious root formation, in different segregating populations of wheat, maize, rice, barley, and barnyard grass, as provided in Table 2.

As multiple waterlogging-related variables were utilized for QTL analysis in these studies, comparing the genetic underpinnings of waterlogging or flooding tolerance among various crops is still challenging. In addition, the lack of shared markers across several genetic linkage maps, and occasionally even between different populations within the same species, makes it difficult to compare QTLs for waterlogging tolerance found in different species. Recently, the multiparent advanced generation intercross (MAGIC) technique has been used to give improved recombination and mapping resolution by multiple alleles introgression in order to reduce the constraints associated with biparental populations [103]. Eight indica parents were intercrossed to create the MAGIC population of rice, which was utilized to find waterlogging tolerance QTLs [104]. In order to map QTLs in maize, a MAGIC population made up of eight genetically distinct lines was compared to the nested association mapping (NAM) population. In comparison to the NAM population, which had a common parentage, the MAGIC population showed a stronger mapping power [105]. Eight spring genotypes of barley were utilized to create a MAGIC population, which was then used to map and characterize the flowering-time gene Vrn-H3 from QFT.MAGIC.HA-7H.A [106]. The majority of QTLs were discovered to be genetic-background-specific or heavily impacted by the environment and G × E interactions, despite the MAGIC population having offered increased genetic diversity and superior resolution for QTL mapping. Several QTLs were discovered under a particular situation, as we have already discussed. This suggests that they should be evaluated under a variety of environmental conditions in order to determine their stability and effect before being considered for use in breeding programs. Consequently, increasing plant waterlogging tolerance through the use of cutting-edge biotechnological technologies could greatly boost plant breeders’ efficiency by providing them with practically valuable molecular markers for waterlogging tolerance. 

Another valuable molecular method for improving waterlogging or flooding tolerance in sustainable agriculture is the overexpression or mutation of target genes. Previously many studies have been conducted to generate waterlogging-resilient crops as well as model plants. For example, overexpression of *Vitreoscilla* hemoglobin (*VHb*) gene in Arabidopsis significantly improved the flooding tolerance by improving various traits, such as root length traits, plant height, and shoot dry weight [107]. Similarly, overexpression of *VHb* gene in Cabbage and Petunias increases waterlogging tolerance [108,109]. According to Raineri et al. [110], overexpression of *HaHB11* in *Zea mays* significantly increased the waterlogging tolerance when compared to wild plants. On the other hand, Arabidopsis plants expressing *AtACO5* gene increased ET accumulation, and cell expansion which in turn modulated key traits leading to waterlogging stress resilience [111]. Interestingly, *CsARN6.1* transgenic cucumber plants show AR formation [112]. Yin et al. [113] generated *PhERF2* petunia transgenic plants which were found to be waterlogging-tolerant when compared to wild plants. In contrast, the RNAi line of *PhERF2* was found to be more vulnerable to flooding stress. Similarly, overexpression of *TaERFVII.1* gene in *Triticum staivum* showed enhanced waterlogging resistance with improved chlorophyll content, higher survival rate, and grain weight [114]. However, *TaERFVII.1* gene silencing leads to reduced expression of waterlogging-tolerant genes and was found to be more sensitive to waterlogging. Previous studies have shown that overexpression of *HvERF2.11* gene in *Arabidopsis* confers waterlogging resistance by increasing the activity of antioxidant enzymes (*AtSOD1*, *AtPOD1*) and expression of ET biosynthetic genes linked with waterlogging tolerance [115]. Cabello et al. [116] developed waterlogging-tolerant *Arabidopsis* plants by overexpressing *HaHB11* and found that transgenic plants showed improved physiological, biochemical, and anatomical traits when compared to wild-type plants [116]. Recently, it was found that overexpression of *ThADH1* and *ThADH4* in Populus confers resilience to waterlogging stress and increases survival rate under low oxygen conditions [117]. Furthermore, we have summarized the role of transgenic technology in improving waterlogging tolerance in different crop systems (Table 3).

### 5.2. Present: Omics Approaches for Understanding Waterlogging Tolerance in Plants

In plant biology, decoding gene functions is crucial to decipher the signaling pathways that regulate plant growth and stress response traits. Although forward genetics has long been used in plant biology to elucidate gene functions, it has many limitations, such as laborious procedure, time consumption, and fractional validations. However, in the last two decades, a variety of new molecular profiling techniques emerged with the introduction of next-generation sequencing, opening the door for massively parallel hypothesis development and reverse genetics validation. With the advent of multiomics, there has been significant progress in decoding the molecular complexity of plant growth and stress responses which paved the way for developing stress and high-yielding cultivars. In this review, we have provided in-depth information on the role of multiomics in waterlogging signaling in plants and highlighted the key points with knowledge gaps. To boost waterlogging stress tolerance in plants, knowledge of stress perception, signal transduction, gene networks, proteins, metabolites, and ions is crucial. The “omics” methods, which basically include genomics, transcriptomics, metabolomics, and proteomics, are used to analyze the above key traits of proteins, metabolites, new genes, and ions involved in waterlogging stress signaling [129]. The creation of NGS and high-throughput genotyping techniques has opened a new area of research and development in plant biology, such as gene mining, marker development, genotyping, highly dense molecular linkage mapping, identification of specific genetic loci, gene tagging, single-base polymorphisms, and identification of transcription factors, that has provided new insights into both breeding and biotechnology-based crop development. Similarly, the application of omics tools has paved the way to understand and decode the molecular intricacy of waterlogging signaling mechanism in plants and also provided novel potential candidate genes for molecular breeding or genetic engineering for the development of waterlogging-resistant crop varieties.

### 5.3. Transcriptional, Metabolic, and Translational Profiling under Waterlogging Stress in Plants

Over the last two decades, various omics approaches have been used to unravel the transcriptional, translational, and metabolic responses in plants after waterlogging stress, which have been instrumental in identifying differential traits in waterlogging-susceptible or -tolerant plants. For instance, Licausi et al. [130] reported 1900 TFs and 180 pri-miRNAs in Arabidopsis plants after being exposed to flooding stress. They also highlighted that these TFs and miRNAs were important regulators of hypoxia-related genes. Previous studies have identified many hypoxia-responsive TFs, such as *ERF*, *NAC*, *MYB*, *ATAF CUC*, and *PHD* families, in different plant systems [130,131,132,133]. In maize, transcriptional profiling during waterlogging stress has revealed many key differential genes associated with cell wall modification, ROS production, calcium signaling, and antioxidant system [134,135,136]. According to a recent study by Cao et al. [137], the majority of the transcription factors (TFs) that are differentially expressed in cassava under waterlogging stress are encoded by genes from the *NAC*, *MYB*, *AP2/ERF*, and *WRKY* families, indicating that these genes are important in waterlogging resilience. Recently, a transcriptome study in pigeon pea plants under waterlogging stress was carried out which revealed a diverse number of differentially expressed genes associated with their physiological, anatomical adaptive traits [138]. Similarly, transcriptome analysis in *Vigna vexillata* roots after waterlogging stress showed many important differentially expressed genes linked with ET biosynthesis, glycolysis, and fermentation [139]. On the other hand, in *Medicago sativa*, many differentially expressed genes related to photosynthesis and nitrogen-metabolism-related genes were identified after waterlogging stress [140]. Transcriptome analysis in *G. max* after waterlogging stress showed a set of differentially expressed genes related to ET signaling, energy metabolism, and fermentation pathways [141]. During waterlogging stress in wheat plants, a transcriptome analysis was performed which showed a number of differentially expressed genes associated with oxidoreductase activity and biological response to ABA and SA [40].

Proteomic studies were also used in different crop systems after waterlogging stress in order to identify differentially expressed proteins. For example, a proteomic study was performed in *Brassica napus* after waterlogging stress which revealed key differentially expressed proteins related to oxidation–reduction process (BnaA09g29780D), response to ethylene (BnaA09g07120D), stress response (BnaC08g02330D), (BnaC02g24210D), and response to JA (BnaC02g24210D) [35]. In another study, Oh et al. [142] performed a proteomic analysis in *G. max* and identified 97 proteins related to waterlogging signaling traits. Similarly, another study has reported diverse proteins such as beta-glucosidase (31) and beta-amylase (5) in G. max after waterlogging stress [143]. On the other hand, proteome analysis of *Sesamum indicum* under waterlogging stress revealed the presence of several proteins, including OEE1, HSPs, Chaperones, and ATPs [144]. In addition, several differentially expressed proteins, including Hsp cognate 70, plastidic cysteine synthase 1, rubisco large/small subunits, rubisco activase, Cytochrome P450, and glycinamide ribonucleotide synthase, were found in Lycopersicon esculentum under waterlogging stress [145]. A comparative proteomic analysis was conducted on barley to find proteins that were differentially expressed in cultivars that were more or less tolerant to flooding. Based on their research, they reported that waterlogging-resistant cultivars have high expression levels of important proteins, such as PDC, ACC oxidase, and GST [60]. The plants under low oxygen concentration lead to fluctuations in development and growth by affecting their carbohydrate and metabolic activity [146,147,148,149]. One of the key areas for enhancing stress resilience is the involvement of metabolites in plant stress resilience. In the past, numerous metabolomics investigations were conducted in various crop systems after waterlogging stress to uncover possible metabolites and their function in waterlogging stress tolerance. For example, in *Medicago truncatula* after waterlogging stress, a metabolomics technique was used which unveiled a greater accumulation of numerous metabolites, such as sugars, organic acid, aromatics, glycine, alanine, glutamine, and lysine, that may play a critical role in stress tolerance [150]. Similarly, in *G. max*, three key metabolites, such as pyruvate, NADH2, and glycine, and gamma-aminobutyric acid, succinate, and citrate were found abundantly after waterlogging stress [151]. On the other hand, a metabolomics investigation on *Helianthus annuus* following waterlogging stress reveals a greater accumulation of a number of metabolites, including alanine, sugars, polyols, aconitate, citrate, and phosphate, all of which may be important for metabolism under waterlogging stress [152]. Furthermore, we have summarized the role of omics to identify key genes/metabolites during waterlogging stress in plants (Table 4).

### 5.4. Future: Integrated Omics and Panomics for Waterlogging Tolerance in Plants

**Figure 3:**
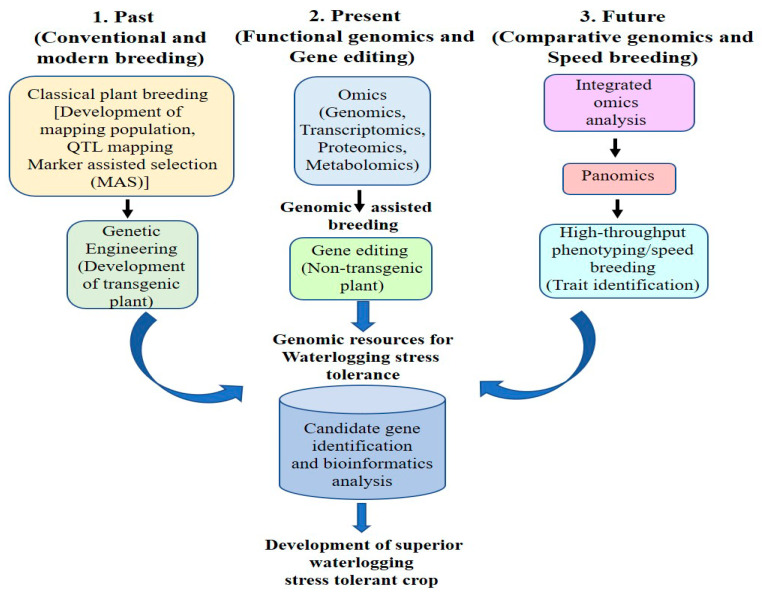
Strategies for improving waterlogging tolerance in plants highlighting (1) Past: conventional and modern breeding approaches (i.e., mapping population and genetic engineering), (2) Present: omics and gene-editing approaches, and (3) Future: comparative genomics (i.e., integrated omics and panomics) and high-throughput phenotyping (speed breeding) approaches. Furthermore, we have presented how the integration of all genomic resources and bioinformatics analysis data would help in the identification of candidate genes for developing a superior cultivar for waterlogging stress tolerance.

Recent years have seen an upsurge in the generation of enormous data from the genome, transcriptome, proteome, metabolome, and other sources under waterlogging stress in a range of plant species. However, because these datasets in several crop species were analyzed independently, a full understanding of the molecular underpinnings of complex features and biological networks was not attainable [157]. Consequently, a systems biology method called “PANOMICS” is necessary to comprehend the flow of biological information underlying complex features. This technique involves the integration of various omics data, prediction of cellular processes, and new modeling [22]. In-depth phenomics and environmental data and their integration with multiomics will also help to better understand the molecular basis of the terroir–phenotype connection. Integration of multiomics data will decrease false positives for genotype–phenotype prediction produced by single data sources [158]. Waterlogging tolerance is a complex process which is relied on various host, environmental, and other traits. Hence, PANOMICS can provide novel insights into deciphering the complexity of waterlogging signaling mechanism and signal transduction (Figure 3). In addition to the methodologies discussed above, artificial intelligence (AI) and deep learning (DL) can also provide novel avenues for phenotypic and proper functional validation of complex waterlogging traits. Currently, the integration of multiomics, artificial intelligence, and deep learning has transformed the area of plant biology and made it simpler for researchers to analyze the most complex phenotypic and genotypic traits [159]. Although there has been a large amount of data generated in different model and nonmodel plants, their functional validation remains the most difficult task for developing future waterlogging smart crops. In this context, the PANOMICS platform and genome-editing technologies will also need to be more closely integrated in order to advance precision breeding for developing waterlogging-resistant cultivars (Figure 3).

## 6. Role of High-Throughput Phenotyping Tool in Waterlogging Stress

Plant phenotyping provides an overall portrait of different plant traits, such as growth, development, seed quality, yield, resistance, and architecture. In plant biology, conventional phenotyping had many demerits in measuring the above traits, such as time-consuming, laborious, less accuracy, low output, and damage to plants [160]. However, many of these limitations have been addressed by the development of high-throughput phenotyping tools, opening up new avenues for research in the fields of functional genomics and plant breeding. High-throughput phenotyping tools integrate various hi-tech tools, such as robotics, computer-based data, spectroscopy, and high-throughput imaging. Although significant progress has been made in creating high-throughput phenotyping methods to screen abiotic stress-tolerant or -sensitive phenotypic traits, there are no standardized phenotyping techniques for waterlogging stress. The development of more precise phenotyping protocols has been greatly hampered by its dependence on numerous environmental conditions and fluctuations during waterlogging stress. Traditional phenotypic scoring during waterlogging stress has been performed by skilled laborers in glasshouse environments (e.g., visual stress scoring) or field environments (e.g., flowering time); however, this procedure is time-consuming and laborious [161]. Phenotyping for waterlogging tolerance has remained challenging due to growth stage, time duration, soil type, and temperature. In this regard, harnessing the potential of high-throughput phenotyping can provide novel avenues for measuring phenotypic traits more accurately and timely during waterlogging (Figure 3). The use of AI in plant phenotyping tools has further advanced this cutting-edge technology for analyzing complex phenotypic traits. In addition, the combination of several waterlogging phenotyping techniques, such as pot and field, as well as the use of various image sensors will produce large datasets and is expected to make it easier and more accurate to characterize the tolerance to waterlogging among crop species.

## 7. Conclusions

Waterlogging is a major concern in modern agriculture since it affects the majority of agriculturally essential crops, resulting in massive production and economic losses. Therefore, it is important to find novel mitigative measures to address this problem. The effect of waterlogging on plant growth and development varies within the same species or within different crops, so do plants’ adaptive responses. Among abiotic stressors, waterlogging signaling mechanism in plants is not fully understood despite the availability of high-throughput tools. For example, how plants sense early waterlogging signals prior to hypoxia remains enigmatic. Secondly, how cell wall sensors or ion channels are involved in perceiving early waterlogging signals and how they translate them into biochemical adaptive responses remains largely unknown. How the plant microbiome interactions are affected during initial flooding or prolonged waterlogging stress is still largely unknown. Nevertheless, there has been significant progress in the morphological and anatomical adaptive responses in plants against waterlogging stress, for example, the formation of ARs, aerenchyma, and metabolic reprogramming such as anaerobic energy production. In spite of that how these processes are driven by different signaling players remains largely unknown. In this regard, harnessing the potential of multiomics, gene editing, and molecular breeding can provide novel insights into deciphering the molecular complexity of waterlogging signaling mechanism and also help in identifying potential gene networks or signaling pathways that can be further utilized for developing future smart waterlogging-resilient crops.

## Figures and Tables

**Table 1:** Effect of waterlogging stress (WS) on different morphological, physiological, and biochemical traits in different model and crop systems.

Species	WS Condition	Affected Traits	References
*Triticum* *aestivum* L.	1 wk	Dry weight of stem and root ↓ Length of root ↓ Ratio of root/shoot ↓ Root aerenchyma ↑	[33]
*Solanum* *dulcamara*	1 wk	Stem region ↓ and adventitious root ↑ (ET↑, ABA↓)	[34]
*Brassica napus* L.	3 d	Length of root and shoot ↓ Fresh weight ↓	[35]
*Glycine max* (L.) Merr. cv. “Williams 82”	10 d	Length of root ↓ Development of lateral root and root hairs ↓	[36]
*Glycine max* L. (S99-2281)	10 d	Length of shoot ↓ Fresh weight of shoot and root ↓ Root aerenchyma ↑ Adventitious root ↑	[37]
*Zea mays* L. (DH605, ZD958)	3 and 6 d	Height of plant and ear ↓ Leaf area index ↓ Yield ↓ Bald tip ↑	[38]
*Triticum* *aestivum* L. (ZM22)	72 h	Germination ↓ Coleoptile height ↓ Amyloplast ↑	[39]
*Hordeum vulgare* L. (Franklin)	21 d	Leaf area ↓ Dry and fresh weight of shoot ↓ Plant height ↓ Total length and number of adventitious root ↑ Leaf aerenchyma ↑ Chlorosis and age of leaf ↑	[40]

**Table 2:** List of identified QTLs related with waterlogging tolerance in different plants.

Species	Mapping Population Type	QTL	Traits	References
Maize	BC3F4, RILs, F2, F2:3	Subtol6, Qarf7.04–7.05, Qarf8.05, sdw9-4, tdw9-2, tdw9-3	Leaf chlorosis, mean leaf senescence score, adventitious root formation, shoot dry weight, total dry weight	[90,91,92,93,94]
Rice	F2	qTIL1 C9285, qTIL1 T65, Sub1, qTIL12 C9285, qTIL12 W0120, qNEI12 C9285, qNEI12 W0120, qLEI12 C9285, qLEI12 W0120	Number and total internode length, green leaf recovery, number of elongated internodes	[95]
Wheat	RILs	QRfbio.ua-1B-WGH, QSfbio.ua-1B-WGH, QSpadpost.ua-1B-WF, QSpad.ua-1D.5, GRI-7A	Shoot and root fresh biomass, chlorophyll content, shoot and root dry biomass, seed germination rate	[96,97]
Barley	DH lines	KWw2.1, GSw1.1/2.1, tfy1.1-1, QWI.YyFr.2H, tfy1.2-1/2.1-1, tfy1.1-2, QWL.YeFr.4H, QTL-AER, QTL-WL-4H, yfy2.2-3, GYw1.2	Kernel weight, grains per spike, leaf chlorosis, plant healthiness, yellow leaf percentage, survival rate, aerenchyma formation, waterlogging tolerance, root porosity, grain yield	[52,78,98,99,100,101,102]

**Table 3:** The role of genetic engineering in developing waterlogging-tolerant plants.

Gene	Transgenic Plant	Gene Source	Waterlogging Tolerance	References
*Pdc1* (pyruvate decarboxylase isozyme 1)	*O. sativa*	*O. sativa*	Enhanced waterlogging tolerance	[118]
*OsSub1A* (ethylene-response-factor-like submergence tolerance gene)	*O. sativa*	*O. sativa*	Enhanced waterlogging tolerance in rice plants by increasing the expression of ADH1	[119,120,121]
*Pdc1* (pyruvate decarboxylase isozyme 1)	*A. thaliana*	*A. thaliana*	Confers waterlogging tolerance	[122]
*Pdc2* (pyruvate decarboxylase isozyme 2)	*A. thaliana*	*A. thaliana*	Enhanced waterlogging tolerance	[122]
*AtACO5* (1-aminocyclopropane-1-carboxylic acid oxidase) and *AtACS* (acetyl-CoA synthetase)	*A. thaliana*	*A. thaliana*	Increased ET levels and waterlogging tolerance	[111]
*AtLDH* (lactate dehydrogenase)	*A. thaliana*	*A. thaliana*	Confers hypoxia tolerance by increasing PDC enzyme activity	[123]
*AtRAP2.6L* (member of ERF subfamily)	*A. thaliana*	*A. thaliana*	Enhanced the activity of antioxidant enzymes and transcript levels of ABA biosynthesis genes, stomatal closure	[68]
*GLB1* class I hemoglobin (Hb)	*A. thaliana*	*Parasponia andersonii*	Enhanced resistance to hypoxia	[124]
Hb (hemoglobin)	*Brassica oleracea*	*Vitreoscilla filiformis*	Confers waterlogging tolerance	[109]
*ACC* (1-aminocyclopropane-1-carboxylic acid) deaminase	*Solanum lycopersicum*	Enterobacter	Confers waterlogging tolerance	[125]
*ipt* (isopentenyl transferase in cytokinin biosynthesis)	*A. thaliana*	*A. thaliana*	Confers waterlogging tolerance	[126,127]
*ZmEREB180* (a group VII ethylene response factor gene)	*Zea mays*	*Zea mays*	Confers waterlogging tolerance by stimulating AR formation	[66]
*AdRAP2.3* (member of ERF subfamily)	*Actinidia deliciosa*	*Nicotiana tabacum*	Enhanced ADH and PDC enzyme activities	[128]
*HvERF2.11* (ethylene responsive factor 2)	*Hordeum vulgare*	*A. thaliana*	Stimulates the expression level of ET genes and also increases antioxidant enzyme activity	[115]
*HaHB11* (homeodomain-leucine zipper I subfamily)	*Helianthus annus*	*A. thaliana*	Confers waterlogging tolerance	[116]
*ThADH1* (alcohol dehydrogenase 1) and *ThADH4* (alcohol dehydrogenase 4)	*Populus alba*	*Taxodium mucronatum* Tenore × *Taxodium distichum* (L.). Rich	Confers waterlogging and hypoxia tolerance	[117]
*HvADH4* (alcohol dehydrogenase 4)	*Hordeum vulgare*	*A. thaliana*	Confers waterlogging tolerance by enhancing the antioxidant enzyme activity	[40]

**Table 4:** Role of omics tools in deciphering waterlogging signaling mechanism in different crop systems.

Omics Study	Species	WS Condition	Key Genes/Metabolites/Proteins	References
Transcriptomics	*Chrysanthemum morifolium* (Nannongxuefeng)	12 h	N-end rule pathway (RAP2.3, HRE2, ATE, PCO1, PCO2) ↑ ROS signaling (POD, AOX1a) ↑ Anaerobic respiration and carbohydrate metabolism (ADH, PDC, SUS1, PDC1) ↑ Hsp 83-like, Chaperone protein ClpB1-like, Snakin-2-like isoform X1 ↑	[153]
	*Actinidia valvata* (KR5)	12, 24, 72 h	ROS scavenging pathway (POD, CAT) ↑, NADH-GOGAT/AlaAT, ERF77 ↑	[154]
	*Manihot esculenta* Grantz	6 d	Photosynthesis, RNA transport, RNA degradation, amino metabolism ↑	[137]
	*T. aestivum* L. (ZM22)	72 h	Oxidoreductase activity, biological response to ABA and SA ↑	[39]
	*Hordeum vulgare* L. (Franklin)	24 h	Metabolic process (biosynthesis of secondary metabolites and phenylpropanoid), transferase activity, catalytic activity ↑	[40]
		72 h	Oxidation–reduction process, protein binding, catalytic activity ↑	
Proteomics	*B. napus* L. (ZS9, tolerant cultivar)	4, 8, 12 h	Oxidation–reduction process (BnaA09g29780D), response to ethylene (BnaA09g07120D) ↑ Abiotic stress response (BnaC08g02330D), (BnaC02g24210D), response to jasmonic acid (BnaC02g24210D) ↓	[35]
	*B. napus* L. (GH01, sensitive cultivar)	4, 8, 12 h	Abiotic stress response (BnaC08g02330D), response to ethylene (BnaA09g07120D) ↑ Oxidation–reduction process (BnaA09g29780D), response to jasmonic acid (BnaC02g24210D) ↓	
	*G. max* L. cultivar Enrei	2 d	Fermentation and glycolysis-related proteins ↑ Degradation/synthesis/posttranslational modification of proteins, hormone/cell wall metabolisms, and DNA synthesis ↓	[142]
	*Sesamum indicum* L., cv. Miryang 44	2, 3 d	Photosynthesis (OEE1), stress defense (HSPs, Chaperones), energy metabolism (ATPs, GS) ↑	[144]
	*Lycopersicon esculentum* L. cv. Koma	24, 48, 72 h	Stress and defense related (Hsp cognate 70, plastidic cysteine synthase1) ↑ Photosynthesis (rubisco large/small subunits, rubisco activase), biosynthesis and metabolism of protein (Cytochrome P450, glycinamide ribonucleotide synthetase) ↓	[145]
Metabolomics	*M. truncatula*	7 and 21 d	Sugars, organic acid, aromatics, glycine, alanine, glutamine, lysine ↑ Nitrogenous compounds, threitol ↓	[150]
	*H. annuus*	2, 7, 14 d	Alanine, sugars, polyols, aconitate, citrate, phosphate ↑ Aspartate, fumarate ↓	[155]
	*Elaeis guineensis*	1, 2, 3, 7 wks	Polyol (myoinositol) ↑ Aconitate, citrate, serine, asparine ↓	[152]
	*G. max* L. cultivar Enrei	2 d	Alanine, AMP, cysteine, DHAP, GABA, glycine ↑ 2-oxoglutarate, acetyl-CoA, allantonin, aspartic acid, fumarate, cinnamate, glutamine ↓	[151]
	*T. aestivum* (Chinese spring)	12 d	Glycine, alanine, GABA ↑ Asparagine, pyruvate ↓	[156]

## Data Availability

Not applicable.
